# A Variational Bayesian Constrained EKF for Sonar-Based Underwater Target Tracking in Shallow Water

**DOI:** 10.3390/s26175591

**Published:** 2026-09-03

**Authors:** Hongkun Zhou, Yunfei Ding, Hanlin Gao, Gang Wang, Tong Ge, Ying Zhang

**Affiliations:** 1 School of Ocean and Civil Engineering, Shanghai Jiao Tong University, Shanghai 200030, China; zhouhongkun@sjtu.edu.cn (H.Z.); tongge@sjtu.edu.cn (T.G.); 2 The Kunming Shipborne Equipment Research and Test Center, Kunming 650025, China; 3 School of Information and Communication Engineering, University of Electronic Science and Technology of China, Chengdu 611731, China; 202612010109@std.uestc.edu.cn (Y.D.); wanggang_hld@uestc.edu.cn (G.W.); 4 School of Mechanical and Electrical Engineering, University of Electronic Science and Technology of China, Chengdu 611731, China; 202511040639@std.uestc.edu.cn; 5 School of Resources and Environment, University of Electronic Science and Technology of China, Chengdu 611731, China

**Keywords:** active sonar, underwater target tracking, shallow water, constrained extended Kalman filter, variational Bayesian filtering, range-dependent covariance, physical constraints, adaptive filtering

## Abstract

Accurate localization of underwater targets in shallow water is challenging because nonlinear sonar geometry, range-amplified angular errors, uncertain measurement noise, and environmental constraints jointly degrade state estimation. This paper proposes a variational Bayesian constrained extended Kalman filter (VB-C-EKF) for active-sonar-based underwater target tracking. A weak-maneuver motion model and an active-sonar range–bearing–elevation–Doppler measurement model are adopted, while bathymetric depth, speed, and reachable-region constraints are incorporated through sequential local Mahalanobis projection with a conservatively regularized covariance correction. To address unknown and time-varying measurement noise, the measurement-noise covariance is recursively estimated using a variational Bayesian scheme with an inverse-Wishart prior and a forgetting mechanism. In Monte Carlo experiments, the proposed method achieved an overall three-dimensional position RMSE of 7.57 m with a 95% confidence-interval half-width of 0.25 m, while maintaining zero depth/speed violations. Its mean normalized innovation squared and normalized estimation error squared were 4.04 and 6.79, respectively, and its average runtime was 0.225 ms per update. These results show that jointly adapting measurement uncertainty and enforcing physical constraints improves accuracy, feasibility, and covariance consistency under the simulated shallow-water conditions.

## 1. Introduction

Active-sonar-based underwater target tracking is important for autonomous underwater sensing, maritime safety monitoring, distributed acoustic surveillance, and dynamic-node navigation in shallow-water environments [1,2,3]. However, shallow-water localization is strongly affected by multipath propagation, seabed and surface reverberation, spatially nonuniform sound-speed profiles, platform motion, and dense false alarms [4,5,6]. Compared with large or highly maneuvering targets, small targets usually have weak acoustic radiation, low target strength, and limited maneuverability, which makes single-frame detection unreliable and makes target intent difficult to infer from a short observation interval [7,8].

Classical target-tracking and navigation-estimation theory provides the basic Bayesian filtering, Kalman filtering, nonlinear filtering, particle filtering, and multiple-model tools for this problem [9,10,11,12,13,14,15].

Recent robust filtering studies further show that non-Gaussian measurement noise, outliers, impulse disturbances, and mixture-distributed trajectory uncertainty should be explicitly considered in practical state estimation systems [16,17,18,19]. Student’s-*t* filtering provides a representative heavy-tailed alternative for suppressing abnormal measurements [20].

In recent years, underwater target detection and tracking have developed along several directions. First, sonar signal processing and learning-based perception have been used to improve weak-target detection. Pulsed active sonar waveform design has been studied for high-speed underwater target detection and tracking [4]. Deep segmentation and forward-looking sonar methods have been applied to weak acoustic target tracking and sonar-image target tracking [5,6]. For sonar images, improved deep detection networks have also been reported to enhance underwater target detection performance [7]. Small-sample tracking methods that combine Siamese networks, Kalman filtering, and reinforcement learning have been proposed to reduce dependence on large labeled sonar datasets [8].

Second, underwater acoustic sensing and networked tracking have received increasing attention. Vector-sensor-array systems have been used for autonomous detection, localization, and tracking of ships by underwater acoustic sensing [1]. Distributed bearing-only target tracking algorithms have been developed for underwater sensor networks with communication and resource constraints [2,21]. Modified adaptive Kalman filtering has been investigated for distributed underwater multi-target passive tracking [22]. Dynamic-node localization and self-powered acoustic sensing further show that underwater localization and tracking are becoming more distributed, asynchronous, and platform-dependent [3,23]. Multi-source fusion, such as AIS and sonar fusion, has also been explored to improve target detection in incomplete and misaligned sensing conditions [24].

Although these studies have improved target detection, sonar perception, and distributed tracking, several issues remain open for active-sonar underwater target tracking in shallow water. Many learning-based methods emphasize detection or image-level tracking and do not explicitly model the physical amplification of angular measurement errors with range. Some networked tracking methods assume a known or calibrated measurement noise covariance, while in real shallow water the covariance varies with range, multipath, reverberation, platform motion, and target aspect. In addition, physically impossible estimates may still appear when the filter tries to explain outlier-contaminated measurements without using depth, speed, and reachable-region constraints.

A particularly important issue is that localization error increases with target range. For a sonar with bearing standard deviation $\sigma_{\theta }$, the lateral position uncertainty approximately satisfies

(1) $$\sigma_{⊥}\approx \rho \sigma_{\theta },$$

where ρ is the target-platform range. Therefore, even a small angular error may produce a large horizontal position error at long range. If this effect is ignored and a fixed Cartesian measurement noise is used, the tracker tends to become overconfident and may produce unstable or physically implausible tracks.

The motivation of this work is to build an active-sonar target-tracking method that is consistent with both sonar measurement physics and underwater operational constraints. The tracker should preserve range-amplified uncertainty instead of hiding it with an artificial fixed Cartesian covariance. It should also use bathymetry, target speed bounds, and reachable-region information to reject physically infeasible states. Finally, because the actual measurement noise variance is difficult to obtain in real time, the filter should adapt its observation covariance from the innovation sequence rather than relying only on a manually specified noise model.

This paper presents a constrained variational Bayesian Kalman filtering framework for this problem. The method has five design principles:

(1) model the weak-maneuver target dynamics in a local Cartesian coordinate frame;
(2) use a nonlinear active-sonar range–bearing–elevation–Doppler measurement model with range-dependent measurement covariance;
(3) introduce depth, speed, and reachable-region constraints as physical prior information;
(4) estimate uncertain measurement covariance online with a variational Bayesian update when the nominal sonar noise model is unreliable;
(5) solve the posterior estimate through sequential local Mahalanobis projection and conservatively regularized covariance correction.

The main contributions are summarized as follows. First, an active-sonar range–bearing–elevation–Doppler measurement model is formulated to explicitly represent range-amplified angular localization error. Second, depth, speed, and reachable-region constraints are incorporated through an implementation-consistent local Mahalanobis state projection with a conservative covariance correction. Third, a variational Bayesian adaptive measurement-covariance update is added to handle unknown time-varying observation noise. Fourth, MATLAB Monte Carlo experiments compare the proposed method with the standard EKF, constrained EKF, VB-EKF, and a constrained Student’s-*t* EKF under uncertain range-dependent noise. Constraint ablation, sensitivity analysis, normalized innovation squared (NIS), normalized estimation error squared (NEES), and per-update runtime are also reported. The EKF form is emphasized because it gives transparent equations and is easy to integrate into an engineering tracking system.

## 2. Materials and Methods

### 2.1. Problem Formulation and Target Dynamic Model

Consider a local East-North-Down coordinate frame. The target state at discrete time *k* is

(2) $$x_{k}={\left[\begin{array}{cccccc} p_{x,k} & p_{y,k} & p_{z,k} & v_{x,k} & v_{y,k} & v_{z,k} \end{array}\right]}^{T},$$

where $p_{k}={\left[p_{x,k},p_{y,k},p_{z,k}\right]}^{T}$ is the target position and $v_{k}={\left[v_{x,k},v_{y,k},v_{z,k}\right]}^{T}$ is the target velocity. The vertical coordinate $p_{z}$ is positive downward.

Let the sonar platform position and velocity be $s_{k}$ and $v_{s,k}$, respectively. The relative position and velocity are

(3) $$d_{k}=p_{k}-s_{k},\;u_{k}=v_{k}-v_{s,k}.$$

The tracking objective is to recursively estimate $x_{k}$ and its covariance $P_{k}$ from noisy sonar measurements while satisfying environmental and kinematic constraints.

#### Target Dynamic Model

For a weak-maneuver underwater target, a nearly constant-velocity model is a practical baseline:

(4) $$x_{k}=F_{k}x_{k-1}+w_{k-1},$$

where $w_{k-1}∼N\left(0,Q_{k-1}\right)$ and

(5) $$F_{k}=\left[\begin{array}{cc} I_{3} & \Delta tI_{3}\\ 0_{3} & I_{3} \end{array}\right].$$

Assuming white acceleration noise with standard deviation $\sigma_{a}$, the process covariance is

(6) $$Q_{k-1}=\sigma_{a}^{2}\left[\begin{array}{cc} \frac{\Delta t^{4}}{4}I_{3} & \frac{\Delta t^{3}}{2}I_{3}\\ \frac{\Delta t^{3}}{2}I_{3} & \Delta t^{2}I_{3} \end{array}\right].$$

The value of $\sigma_{a}$ should be small for a low-maneuver target, but not zero. A nonzero process noise allows the filter to absorb unmodeled current disturbance, small heading changes, and acoustic measurement bias.

If the target occasionally performs evasive turns, the model in Equation (4) can be embedded into an IMM filter. The model set may include a constant-velocity model, a constant-acceleration model, and a coordinated-turn model. For the low-maneuver case, the constant-velocity model should have the largest prior probability.

### 2.2. Sonar Measurement and Constraint Modeling

#### 2.2.1. Active-Sonar Range–Bearing–Elevation–Doppler Measurement

The range is

(7) $$\rho_{k}=∥d_{k}∥.$$

The bearing and elevation are

(8) $$\theta_{k}=atan2\left(d_{y,k},d_{x,k}\right),$$

(9) $$\phi_{k}=atan2\left(d_{z,k},\sqrt{d_{x,k}^{2}+d_{y,k}^{2}}\right).$$

The radial velocity measured by Doppler is

(10) $${\dot{\rho }}_{k}=\frac{d_{k}^{T}u_{k}}{\rho_{k}}.$$

Thus, the active-sonar range–bearing–elevation–Doppler measurement model used in this study can be written as

(11) $$z_{k}=h\left(x_{k}\right)+r_{k},$$

where

(12) $$h\left(x_{k}\right)={\left[\begin{array}{cccc} \rho_{k} & \theta_{k} & \phi_{k} & {\dot{\rho }}_{k} \end{array}\right]}^{T}$$

for active-sonar range–bearing–elevation–Doppler observations, and $r_{k}∼N\left(0,R_{k}\right)$.

#### 2.2.2. Range-Dependent Measurement Covariance

The measurement covariance should reflect the fact that angular errors lead to larger Cartesian errors at longer range. In polar/spherical measurement coordinates, one may write

(13) $$R_{k}=\mathrm{diag}\left(\sigma_{\rho ,k}^{2},\sigma_{\theta ,k}^{2},\sigma_{\phi ,k}^{2},\sigma_{\dot{\rho },k}^{2}\right).$$

If the measurement is converted into a Cartesian pseudo-measurement, the covariance should be propagated as

(14) $$R_{p,k}^{c}=J_{c,k}R_{p,k}^{s}J_{c,k}^{T},$$

where $R_{p,k}^{s}$ is the covariance of ${\left[\rho ,\theta ,\phi \right]}^{T}$ and $J_{c,k}$ is the Jacobian of the spherical-to-Cartesian mapping. Equation (14) naturally produces terms proportional to $\rho_{k}^{2}\sigma_{\theta ,k}^{2}$ and $\rho_{k}^{2}\sigma_{\phi ,k}^{2}$.

Equivalently, under the small-angle approximation, the range-amplified position uncertainty can be expressed as

(15) $$\sigma_{⊥,k}\approx \rho_{k}\sigma_{\theta ,k},\;\sigma_{z,\mathrm{ang},k}\approx \rho_{k}\sigma_{\phi ,k}.$$

Thus the long-range tracking condition is not represented by a fixed Cartesian noise level. Instead, the effective cross-range and elevation-induced position errors grow linearly with $\rho_{k}$, and their variances grow quadratically with $\rho_{k}$.

When EKF directly uses the nonlinear angular measurement, the same physical effect appears in the Jacobian. For bearing,

(16) $$\frac{\partial \theta }{\partial p_{x}}=-\frac{d_{y}}{d_{x}^{2}+d_{y}^{2}},\;\frac{\partial \theta }{\partial p_{y}}=\frac{d_{x}}{d_{x}^{2}+d_{y}^{2}}.$$

The sensitivity of bearing to position decreases as range increases, so the posterior covariance remains large along poorly observed directions.

#### 2.2.3. Constraint Modeling

Let the feasible state set be

(17) $$\Omega_{k}=\Omega_{d,k}\cap \Omega_{v,k}\cap \Omega_{r,k},$$

where $\Omega_{d,k}$ is the bathymetric depth constraint, $\Omega_{v,k}$ is the speed constraint, and $\Omega_{r,k}$ is the reachable-region constraint.

##### Bathymetric Depth Constraint

Let $H_{\mathrm{sea}}\left(p_{x},p_{y}\right)$ denote the local water depth obtained from a bathymetric map. With the down-positive vertical axis, the target depth must satisfy

(18) $$d_{\min}\leq p_{z}\leq H_{\mathrm{sea}}\left(p_{x},p_{y}\right)-d_{b},$$

where $d_{\min}$ is the minimum operating depth and $d_{b}$ is a bottom safety margin. The lower and upper bounds remove estimates that are above the surface or inside the seabed.

The nonlinear upper constraint can be written as

(19) $$g_{d}\left(x_{k}\right)=p_{z,k}-H_{\mathrm{sea}}\left(p_{x,k},p_{y,k}\right)+d_{b}\leq 0.$$

For EKF-based projection, it can be locally linearized around an unconstrained estimate $x_{k}^{u}$:

(20) $$g_{d}\left(x_{k}^{u}\right)+G_{d,k}\left(x_{k}-x_{k}^{u}\right)\leq 0,$$

where

(21) $$G_{d,k}={\left[-\frac{\partial H_{\mathrm{sea}}}{\partial p_{x}},-\frac{\partial H_{\mathrm{sea}}}{\partial p_{y}},1,0,0,0\right]}_{x_{k}^{u}}.$$

##### Speed Constraint

Small underwater targets usually have bounded speed. The constraint is

(22) $$v_{\min}\leq ∥v_{k}∥\leq v_{\max}.$$

The upper bound is often more useful:

(23) $$g_{v}\left(x_{k}\right)=∥v_{k}∥-v_{\max}\leq 0.$$

Linearizing Equation (23) around $x_{k}^{u}$ gives

(24) $$g_{v}\left(x_{k}^{u}\right)+G_{v,k}\left(x_{k}-x_{k}^{u}\right)\leq 0,$$

with

(25) $$G_{v,k}=\left[\begin{array}{cccccc} 0 & 0 & 0 & \frac{v_{x}^{u}}{∥v^{u}∥} & \frac{v_{y}^{u}}{∥v^{u}∥} & \frac{v_{z}^{u}}{∥v^{u}∥} \end{array}\right].$$

An acceleration bound may also be used:

(26) $$\frac{∥v_{k}-v_{k-1}^{+}∥}{\Delta t}\leq a_{\max}.$$

##### Reachable-Region Constraint

The reachable region limits the target position at time *k* according to the previous estimate and target speed:

(27) $$∥p_{k}-p_{k-1}^{+}∥\leq v_{\max}\Delta t+\epsilon_{r},$$

where $\epsilon_{r}$ allows process noise and environmental current. If a navigation channel, restricted area, or bathymetric passability map is available, the reachable set can be refined as

(28) $$p_{k}\in A_{k},$$

where $A_{k}$ denotes the physically reachable and operationally possible area. The reachable-region constraint is especially useful in bearing-only or long-range tracking because it suppresses unrealistic jumps along the line of sight [25].

### 2.3. Constrained EKF Solution and Implementation Procedure

#### 2.3.1. Unconstrained Prediction and Update

The EKF prediction is

(29) $${\hat{x}}_{k}^{-}=F_{k}{\hat{x}}_{k-1}^{+},$$

(30) $$P_{k}^{-}=F_{k}P_{k-1}^{+}F_{k}^{T}+Q_{k-1}.$$

Let $H_{k}$ be the Jacobian of h(x) at ${\hat{x}}_{k}^{-}$. The innovation is

(31) $$\nu_{k}=z_{k}-h\left({\hat{x}}_{k}^{-}\right).$$

Angular components of $\nu_{k}$ must be wrapped to [−π,π]. The innovation covariance and Kalman gain are

(32) $$S_{k}=H_{k}P_{k}^{-}H_{k}^{T}+R_{k},$$

(33) $$K_{k}=P_{k}^{-}H_{k}^{T}S_{k}^{-1}.$$

The unconstrained posterior is

(34) $${\hat{x}}_{k}^{u}={\hat{x}}_{k}^{-}+K_{k}\nu_{k}.$$

The Joseph covariance update is recommended for numerical robustness:

(35) $$\begin{array}{cc} P_{k}^{u}= & \left(I-K_{k}H_{k}\right)P_{k}^{-}{\left(I-K_{k}H_{k}\right)}^{T}\\ & +K_{k}R_{k}K_{k}^{T}. \end{array}$$

#### 2.3.2. Covariance-Weighted Constraint Projection

After the unconstrained update, the estimate is corrected using the feasible set. This follows the standard idea of incorporating nonlinear state constraints into Kalman filtering through constrained estimation or projection [26]. The ideal hard-constrained estimate can be written as

(36) $${\hat{x}}_{k}^{+}=\arg\min_{x\in \Omega_{k}}{\left(x-{\hat{x}}_{k}^{u}\right)}^{T}{\left(P_{k}^{u}\right)}^{-1}\left(x-{\hat{x}}_{k}^{u}\right).$$

This projection chooses the feasible state that is closest to the unconstrained posterior in the Mahalanobis metric. Therefore, highly uncertain components can be corrected more strongly than well-determined components.

If the active constraints are approximated by linear equalities

(37) $$A_{a,k}x=b_{a,k},$$

then the closed-form projection is

(38) $$\begin{array}{c} {\hat{x}}_{k}^{+}={\hat{x}}_{k}^{u}-P_{k}^{u}A_{a,k}^{T}{\left(A_{a,k}P_{k}^{u}A_{a,k}^{T}\right)}^{-1}\left(A_{a,k}{\hat{x}}_{k}^{u}-b_{a,k}\right). \end{array}$$

The projected covariance is

(39) $$\begin{array}{c} P_{k}^{+}=P_{k}^{u}-P_{k}^{u}A_{a,k}^{T}{\left(A_{a,k}P_{k}^{u}A_{a,k}^{T}\right)}^{-1}A_{a,k}P_{k}^{u}. \end{array}$$

For an inequality $g_{j}\left(x\right)\leq 0$, only violated constraints are activated. At sequential projection iteration *j*, the implementation evaluates $g_{j}\left(x^{\left(j\right)}\right)$ and its row Jacobian $A_{j}=\nabla g_{j}{\left(x^{\left(j\right)}\right)}^{T}$.

The local Mahalanobis projection of the state mean is

(40) $$x^{\left(j+1\right)}=x^{\left(j\right)}-P_{k}^{u}A_{j}^{T}{\left(A_{j}P_{k}^{u}A_{j}^{T}\right)}^{-1}g_{j}\left(x^{\left(j\right)}\right).$$

Equation (40) is the closed-form solution for the boundary of the locally linearized constraint; unlike component clipping or Euclidean radial scaling, it accounts for correlations and unequal uncertainty among state components. The nonlinear depth, speed, and reachable-region constraints are relinearized for up to eight passes, with a small feasibility tolerance used to stop the iteration.

Applying an exact hard covariance projection every time a nonlinear boundary is touched can make the recursive filter unrealistically overconfident. The implementation therefore projects the mean to the local boundary using Equation (40), but applies a conservative regularized correction to the covariance. For a final active constraint row $A_{a,k}$, the regularized gain is

(41) $$L_{a,k}=P_{k}^{\left(a\right)}A_{a,k}^{T}{\left(A_{a,k}P_{k}^{\left(a\right)}A_{a,k}^{T}+\varepsilon_{a,k}\right)}^{-1},$$

and the covariance update is

(42) $$\begin{array}{cc} P_{k}^{\left(a+1\right)}= & \left(I-L_{a,k}A_{a,k}\right)P_{k}^{\left(a\right)}{\left(I-L_{a,k}A_{a,k}\right)}^{T}\\ & +L_{a,k}\varepsilon_{a,k}L_{a,k}^{T}. \end{array}$$

Here the regularization variance is

(43) $$\varepsilon_{a,k}=\kappa_{a}A_{a,k}P_{k}^{\left(a\right)}A_{a,k}^{T},$$

Thus, the state mean satisfies the locally linearized physical boundary, while the covariance receives only a conservative correction with gain scaled by $1/(1+\kappa_{a})$. Recommended values is $\kappa_{a}=25$, which retains only 1/26≈3.85% of the covariance reduction produced by an exact hard covariance projection. This setting was adopted to prevent repeated nonlinear boundary contacts from making the filter overconfident, while the state mean is still projected onto the feasible boundary. Increasing $\kappa_{a}$ makes the covariance correction more conservative, whereas decreasing it moves the covariance closer to the exact hard-projection result. Each activated constraint family updates the covariance at most once per time step. This choice was made to prevent the repeated nonlinear projection from producing an artificially small covariance; it is not presented as an exact conditional covariance. Equations (40)–(43) match the MATLAB implementation.

#### 2.3.3. Soft-Constraint Alternative

When the environmental prior is uncertain, constraints can be treated as pseudo-measurements rather than hard constraints. For example, a soft depth constraint can be written as

(44) $$z_{d,k}=0=g_{d}\left(x_{k}\right)+e_{d,k},$$

where $e_{d,k}∼N\left(0,\sigma_{d}^{2}\right)$. A smaller $\sigma_{d}$ enforces a stronger constraint. This form is useful when the bathymetric map has limited resolution or uncertain registration.

#### 2.3.4. Variational Bayesian Adaptive Measurement Covariance

In practice, the true measurement covariance $R_{k}$ is difficult to know in real time because reverberation, platform motion, sound-speed mismatch, and target aspect changes vary with time. A fixed or overly optimistic range-dependent covariance may make the filter overconfident. To address this, a variational Bayesian (VB) adaptive update can be added before the constraint projection [27].

Let $R_{0,k}$ be the nominal range-dependent covariance obtained from the predicted geometry. The VB form treats the unknown covariance as a random matrix with an inverse-Wishart (IW) prior:

(45) $$R_{k}∼\mathrm{IW}\left(\nu_{k},U_{k}\right),$$

where $\nu_{k}$ is the degree-of-freedom parameter and $U_{k}$ is the scale matrix. For online tracking, the prior mean is blended with the previous adaptive estimate:

(46) $${\bar{R}}_{k}=\lambda {\hat{R}}_{k-1}+\left(1-\lambda \right)R_{0,k},\;0<\lambda <1.$$

A larger λ assigns more weight to the previous covariance estimate, resulting in smoother covariance evolution but slower adaptation to rapid noise variations. Conversely, a smaller λ places more emphasis on the current nominal covariance, enabling faster adaptation at the expense of weaker temporal smoothing. In all experiments, the forgetting factor was fixed at λ=0.85.

For a measurement dimension $m_{z}$, choose $\nu_{0}>m_{z}+1$ and set

(47) $$U_{0,k}=\left(\nu_{0}-m_{z}-1\right){\bar{R}}_{k}.$$

During VB iteration *i*, the EKF update is computed with the current covariance estimate ${\hat{R}}_{k}^{\left(i-1\right)}$:

(48) $$S_{k}^{\left(i\right)}=H_{k}P_{k}^{-}H_{k}^{T}+{\hat{R}}_{k}^{\left(i-1\right)},$$

(49) $$K_{k}^{\left(i\right)}=P_{k}^{-}H_{k}^{T}{\left(S_{k}^{\left(i\right)}\right)}^{-1}.$$

This gives ${\hat{x}}_{k}^{\left(i\right)}$ and $P_{k}^{\left(i\right)}$. The nonlinear residual is

(50) $$e_{k}^{\left(i\right)}=z_{k}-h\left({\hat{x}}_{k}^{\left(i\right)}\right),$$

with angular components wrapped to [−π,π]. In the diagonal covariance implementation used here, the covariance update is

(51) $$\begin{array}{c} {\hat{R}}_{k}^{\left(i\right)}=\mathrm{diag}\left(\frac{\mathrm{diag}\left[U_{0,k}+e_{k}^{\left(i\right)}{\left(e_{k}^{\left(i\right)}\right)}^{T}+H_{k}P_{k}^{\left(i\right)}H_{k}^{T}\right]}{\nu_{0}-m_{z}}\right). \end{array}$$

The diagonal form preserves the physical meaning of range, bearing, elevation, and Doppler noise variances and avoids unstable cross-covariance estimates from a short innovation sequence. After several VB iterations, the final unconstrained estimate is projected onto $\Omega_{k}$ using Equation (40). Thus, VB handles uncertain observation noise, while the physical projection handles infeasible states.

#### 2.3.5. Student’s t Robust Comparison Filter

To provide a competitive heavy-tailed baseline, an iteratively reweighted Student’s-*t* constrained EKF (ST-C-EKF) is implemented following the scale-mixture principle of Student’s-*t* filtering [20]. It uses the same dynamic model, nominal covariance $R_{0,k}$, and physical projection as the proposed method. At iteration *i*, define the expected normalized residual energy as

(52) $$\begin{array}{cc} \delta_{k}^{\left(i\right)}= & {\left(e_{k}^{\left(i\right)}\right)}^{T}R_{0,k}^{-1}e_{k}^{\left(i\right)}\\ & +\mathrm{tr}\left[R_{0,k}^{-1}H_{k}P_{k}^{\left(i\right)}H_{k}^{T}\right]. \end{array}$$

The latent precision weight and effective measurement covariance are

(53) $$\omega_{k}^{\left(i\right)}=\min\left\{1,\max\left\{\omega_{\min},\frac{\nu_{t}+m_{z}}{\nu_{t}+\delta_{k}^{\left(i\right)}}\right\}\right\},$$

(54) $$R_{t,k}^{\left(i\right)}=\frac{R_{0,k}}{\omega_{k}^{\left(i\right)}}.$$

Large residuals therefore reduce $\omega_{k}^{\left(i\right)}$ and inflate the measurement covariance. The experiments use $\nu_{t}=4$, $\omega_{\min}=0.05$, and four iterations. Unlike VB-C-EKF, ST-C-EKF performs instantaneous heavy-tail downweighting and does not recursively learn a time-varying covariance.

#### 2.3.6. Implementation Procedure

The complete filtering procedure is summarized in Table 1. The method is compatible with single-station tracking, multi-station fusion, and track-before-detect front ends. In a multi-station case, the measurement vector and Jacobian are formed by stacking the measurements and Jacobians from all sonar platforms.

#### 2.3.7. Stability Analysis

The proposed filter contains three stabilizing mechanisms: the EKF covariance recursion, the local Mahalanobis mean projection with conservative covariance correction, and the VB covariance adaptation. The following analysis gives sufficient local conditions for bounded estimation error. Let the estimation error after projection be

(55) $${\tilde{x}}_{k}^{+}=x_{k}-{\hat{x}}_{k}^{+}.$$

Around the predicted estimate, the EKF error dynamics can be written in linearized form as

(56) $$\begin{array}{cc} {\tilde{x}}_{k}^{u}= & \left(I-K_{k}H_{k}\right)F_{k}{\tilde{x}}_{k-1}^{+}\\ & +\left(I-K_{k}H_{k}\right)w_{k-1}-K_{k}r_{k}+\eta_{k}, \end{array}$$

where $\eta_{k}$ denotes the higher-order linearization remainder. If $F_{k}$ and $H_{k}$ are bounded, the linearized system is uniformly detectable, the process-noise channels provide the required stabilizability, and the covariance matrices satisfy

(57) $$0⪯Q_{k}⪯q_{\max}I,\;r_{\min}I⪯{\hat{R}}_{k}⪯r_{\max}I,$$

then the innovation covariance $S_{k}$ remains positive definite and the Kalman gain is bounded. Under the standard local EKF conditions, including bounded Jacobians, local observability, bounded covariance matrices, and sufficiently accurate first-order linearization within the neighborhood of the estimated trajectory, the posterior error remains locally mean-square bounded.

For an exact metric projection onto a locally convex feasible set, the projection is non-expansive in the covariance metric when the true state is feasible. For any $x_{k}\in \Omega_{k}$, the ideal projection satisfies

(58) $$\begin{array}{cc} \; & {\left(x_{k}-{\hat{x}}_{k}^{+}\right)}^{T}{\left(P_{k}^{u}\right)}^{-1}\left(x_{k}-{\hat{x}}_{k}^{+}\right)\\ \leq & {\left(x_{k}-{\hat{x}}_{k}^{u}\right)}^{T}{\left(P_{k}^{u}\right)}^{-1}\left(x_{k}-{\hat{x}}_{k}^{u}\right). \end{array}$$

This relation should be interpreted as a property of the ideal local projection rather than a global guarantee for the implemented nonlinear recursion. The bathymetric boundary is nonlinear, the constraints are applied sequentially, and the covariance correction is deliberately conservative. If the constraints contain the true state and the local linearization is accurate, the mean projection suppresses infeasible components without treating the sonar measurement itself as more accurate. This property is especially important at long range, where the measurement geometry leaves weakly observable directions.

The VB update contributes to stability by preventing the filter from using an unrealistically small measurement covariance. In the diagonal implementation, the covariance estimate is clipped between lower and upper bounds around the nominal covariance:

(59) $$\alpha_{\min}\mathrm{diag}\left(R_{0,k}\right)\leq \mathrm{diag}\left({\hat{R}}_{k}\right)\leq \alpha_{\max}\mathrm{diag}\left(R_{0,k}\right),$$

where $0<\alpha_{\min}<1<\alpha_{\max}$. Together with $\nu_{0}>m_{z}+1$ in the inverse-Wishart prior, Equation (59) keeps ${\hat{R}}_{k}$ positive definite and prevents the Kalman gain from becoming excessively large under outliers or underestimated angular noise. Hence, VB adaptation improves robustness to unknown range-dependent noise while preserving the bounded-covariance condition in Equation (57).

The analysis therefore states local sufficient conditions rather than a global stability theorem for the nonlinear implementation. The analysis is local and conditional on the validity of the first-order EKF approximation along the estimated trajectory. It assumes a feasible target trajectory, correct data association, bounded process disturbance, bounded covariance recursion, and moderate linearization error. Severe false associations or an infeasible constraint set can still destabilize the tracker and should be handled by gating, track management, and constraint consistency checks.

## 3. Results

A MATLAB Monte Carlo experiment was designed to verify the proposed constrained EKF and the VB adaptive extension. The simulation considers one stationary sonar platform located at the origin and one weak-maneuver underwater target moving in a shallow-water region. The target state is generated by a nearly constant-velocity model with small sinusoidal acceleration disturbances. The sonar measurement includes range, bearing, elevation, and Doppler.

To reflect the fact that the actual observation noise variance is difficult to obtain in real time, the true measurement noise used to generate data is allowed to vary with range and an unknown environmental scale:

(60) $$\begin{array}{cc} g_{k}= & 1+0.35\sin(0.055k+0.4)+0.18\cos(0.019k)\\ & +0.25\min(\rho_{k}/1600,1.5), \end{array}$$

(61) $$\gamma_{k}=\min\left\{1.85,\max\left(0.75,g_{k}\right)\right\}.$$

The true standard deviations are

(62) $$\left[\begin{array}{c} \sigma_{\rho ,k}^{\mathrm{true}}\\ \sigma_{\theta ,k}^{\mathrm{true}}\\ \sigma_{\phi ,k}^{\mathrm{true}}\\ \sigma_{\dot{\rho },k}^{\mathrm{true}} \end{array}\right]=\gamma_{k}\left[\begin{array}{c} 6+0.007\rho_{k}\\ 0.70^{\circ }\\ 0.50^{\circ }\\ 0.20\;m/s \end{array}\right].$$

The filters do not receive Equation (62). Their nominal covariance is built from

(63) $$\left[\begin{array}{c} \sigma_{\rho ,k}^{0}\\ \sigma_{\theta ,k}^{0}\\ \sigma_{\phi ,k}^{0}\\ \sigma_{\dot{\rho },k}^{0} \end{array}\right]=\left[\begin{array}{c} 5+0.0055\rho_{k}\\ 0.55^{\circ }\\ 0.40^{\circ }\\ 0.16\;m/s \end{array}\right].$$

According to Equation (15), when $\gamma_{k}=1$ and $\rho_{k}=1200\;m$, the one-sigma cross-range and elevation-induced position errors are approximately 14.7m and 10.5m, respectively. A 4% outlier probability is introduced using an additive Bernoulli–Gaussian contamination model. Specifically, the simulated measurement is generated as

(64) $$z_{k}=h\left(x_{k}\right)+r_{k}+b_{k}o_{k},$$

where $b_{k}∼\mathrm{Bernoulli}\left(p_{o}\right)$ with $p_{o}=0.04$. The nominal measurement noise $r_{k}$ is Gaussian, where the additional outlier term is generated independently as $o_{k}∼N\left(0,\mathrm{diag}\left[30^{2},{\left(2.8\pi /180\right)}^{2},{\left(1.8\pi /180\right)}^{2},0.8^{2}\right]\right).$

The bathymetric map used in the simulation is

(65) $$H_{\mathrm{sea}}\left(p_{x},p_{y}\right)=33+4\sin\left(p_{x}/600\right)+3\cos\left(p_{y}/500\right).$$

The minimum depth is 3m, the bottom safety margin is 3m, the maximum target speed is 7.2m/s, and the reachable-region bound is constructed from $v_{\max}\Delta t$ with an uncertainty-dependent margin. The sampling interval is 1s, the simulation length is 160s, and 100 Monte Carlo runs are used for the main comparison, constraint ablation, and sensitivity experiments.

Five filters are compared. The first four preserve the two-factor VB/constraint ablation, while the fifth provides a competitive heavy-tailed robust baseline:

- Standard EKF: nonlinear sonar measurement update using the nominal covariance in Equation (63), but without physical constraints.
- Constrained EKF: the same nominal-covariance EKF followed by sequential projection onto the depth, speed, and reachable-region constraints.
- VB-EKF: VB online adaptation of the measurement covariance, but without physical constraints.
- ST-Constrained EKF: Student’s-*t* residual reweighting followed by the same physical projection as the proposed method.
- VB-Constrained EKF: the constrained EKF with VB online adaptation of the measurement covariance.

For Monte Carlo run m=1,…,M and time step k=1,…,K, let $p_{m,k}$ be the true position and ${\hat{p}}_{m,k}^{f}$ be the position estimate of filter *f*. The position error is

(66) $$e_{m,k}^{f}={\hat{p}}_{m,k}^{f}-p_{m,k}={\left[\begin{array}{ccc} e_{x,m,k}^{f} & e_{y,m,k}^{f} & e_{z,m,k}^{f} \end{array}\right]}^{T}.$$

The overall 3D, horizontal, and depth results are the means of the run-wise RMSE values:

(67) $${\mathrm{RMSE}}_{3D}^{f}=\frac{1}{M}\sum_{m=1}^{M}\sqrt{\frac{1}{K}\sum_{k=1}^{K}{∥e_{m,k}^{f}∥}^{2}},$$

(68) $${\mathrm{RMSE}}_{h}^{f}=\frac{1}{M}\sum_{m=1}^{M}\sqrt{\frac{1}{K}\sum_{k=1}^{K}\left[{\left(e_{x,m,k}^{f}\right)}^{2}+{\left(e_{y,m,k}^{f}\right)}^{2}\right]},$$

(69) $${\mathrm{RMSE}}_{z}^{f}=\frac{1}{M}\sum_{m=1}^{M}\sqrt{\frac{1}{K}\sum_{k=1}^{K}{\left(e_{z,m,k}^{f}\right)}^{2}}.$$

The reported RMSE is then obtained by averaging these M=100 run-wise RMSE values. Accordingly, the 95% confidence-interval half-width is computed as $1.96s_{\mathrm{run}}/\sqrt{M}$, and $s_{\mathrm{run}}$ is the sample standard deviation of the 100 run-wise RMSE values.

The long-range metrics use the subset

(70) $$L=\{k|\rho_{k}>1200\;m\},$$

which matches the threshold used in the simulation script. For example, the long-range 3D RMSE is

(71) $${\mathrm{RMSE}}_{3D,L}^{f}=\frac{1}{M}\sum_{m=1}^{M}\sqrt{\frac{1}{\left|L\right|}\sum_{k\in L}{∥e_{m,k}^{f}∥}^{2}},$$

and the long-range horizontal and depth RMSEs are obtained by replacing the squared error term with the corresponding horizontal or depth error term above. The final-time 3D RMSE is

(72) $${\mathrm{RMSE}}_{3D,K}^{f}=\frac{1}{M}\sum_{m=1}^{M}∥e_{m,K}^{f}∥.$$

The reported bound-violation rate counts the depth and speed bound violations used in the simulation. Define

(73) $${\hat{H}}_{m,k}^{f}=H_{\mathrm{sea}}\left({\hat{p}}_{x,m,k}^{f},{\hat{p}}_{y,m,k}^{f}\right).$$

The violation indicator is

(74) Im,kf=1,p^z,m,kf<dminorp^z,m,kf>H^m,kf−db orv^m,kf>vmax,0,otherwise.

Thus,

(75) $$r_{\mathrm{vio}}^{f}=\frac{100}{MK}\sum_{m=1}^{M}\sum_{k=1}^{K}I_{m,k}^{f}\;\left(\%\right).$$

For the main comparison, the table reports the Monte Carlo mean together with the 95% confidence-interval half-width computed from the run-wise metric values as $1.96s/\sqrt{M}$, where *s* is the sample standard deviation across Monte Carlo runs.

Filter consistency is evaluated using NIS and NEES. For filter *f*,

(76) $${\mathrm{NIS}}_{m,k}^{f}={\left(\nu_{m,k}^{f}\right)}^{T}{\left(S_{m,k}^{f}\right)}^{-1}\nu_{m,k}^{f},$$

(77) $${\mathrm{NEES}}_{m,k}^{f}={\left(x_{m,k}-{\hat{x}}_{m,k}^{f}\right)}^{T}{\left(P_{m,k}^{f}\right)}^{-1}\left(x_{m,k}-{\hat{x}}_{m,k}^{f}\right).$$

At each time step, the ensemble averages ANIS and ANEES are formed over the *M* runs. Their two-sided 95% consistency intervals are $\left[\chi_{0.025,Mm_{z}}^{2}/M,\chi_{0.975,Mm_{z}}^{2}/M\right]$ and $\left[\chi_{0.025,Mn_{x}}^{2}/M,\chi_{0.975,Mn_{x}}^{2}/M\right]$, respectively. With M=100, $m_{z}=4$, and $n_{x}=6$, these intervals are [3.465,4.573] and [5.340,6.698]. The reported coverage is the percentage of the 160 time steps for which the ensemble statistic lies inside its interval.

**Figure 1:**
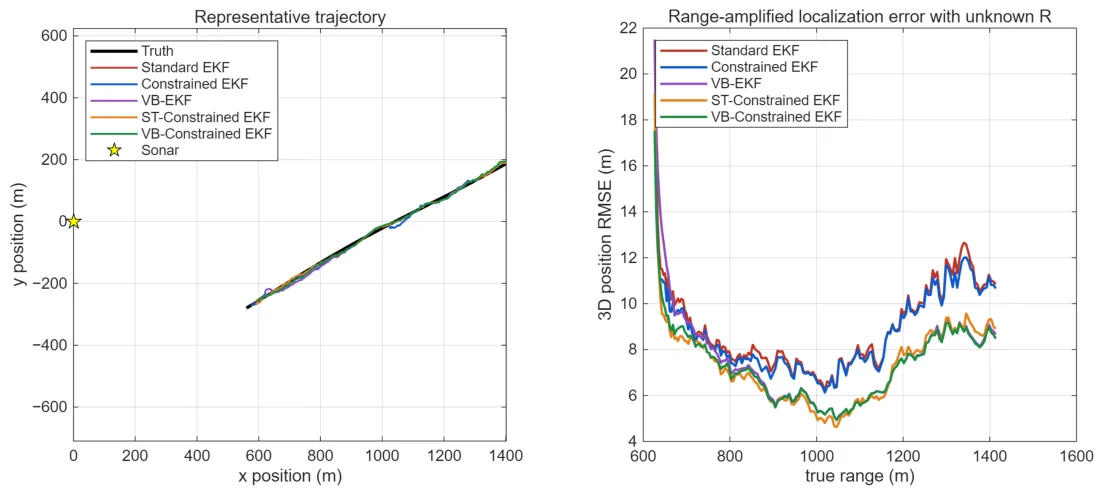
MATLAB simulation results under unknown time-varying measurement covariance. The left panel shows one representative trajectory. The right panel shows the Monte Carlo 3D position RMSE as a function of target range for the standard EKF, constrained EKF, VB-EKF, ST-Constrained EKF, and VB-Constrained EKF.

**Figure 2:**
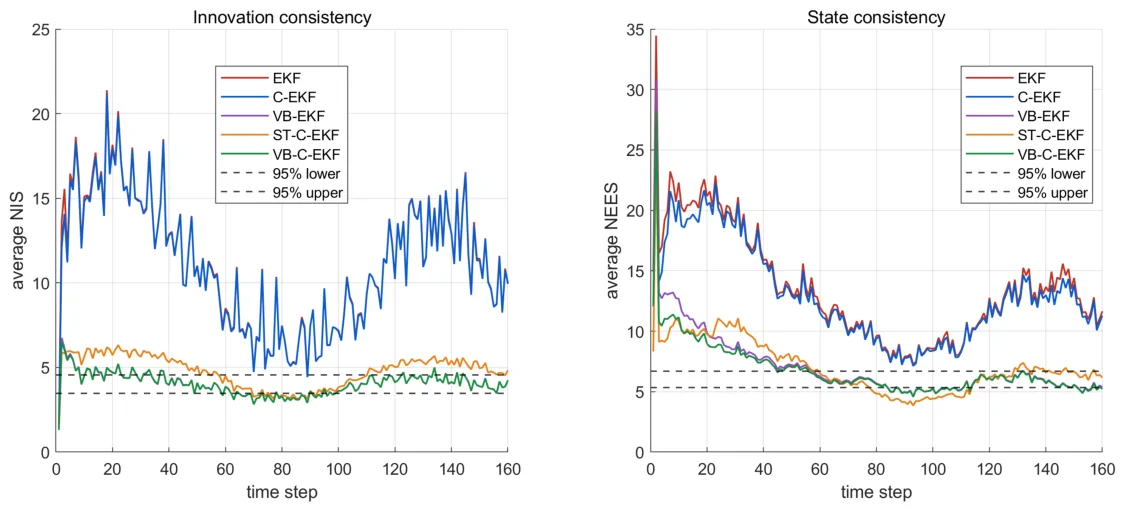
Ensemble-averaged NIS and NEES over time for the five filters. Dashed lines show the corresponding two-sided 95% chi-square consistency bounds.

**Figure 3:**
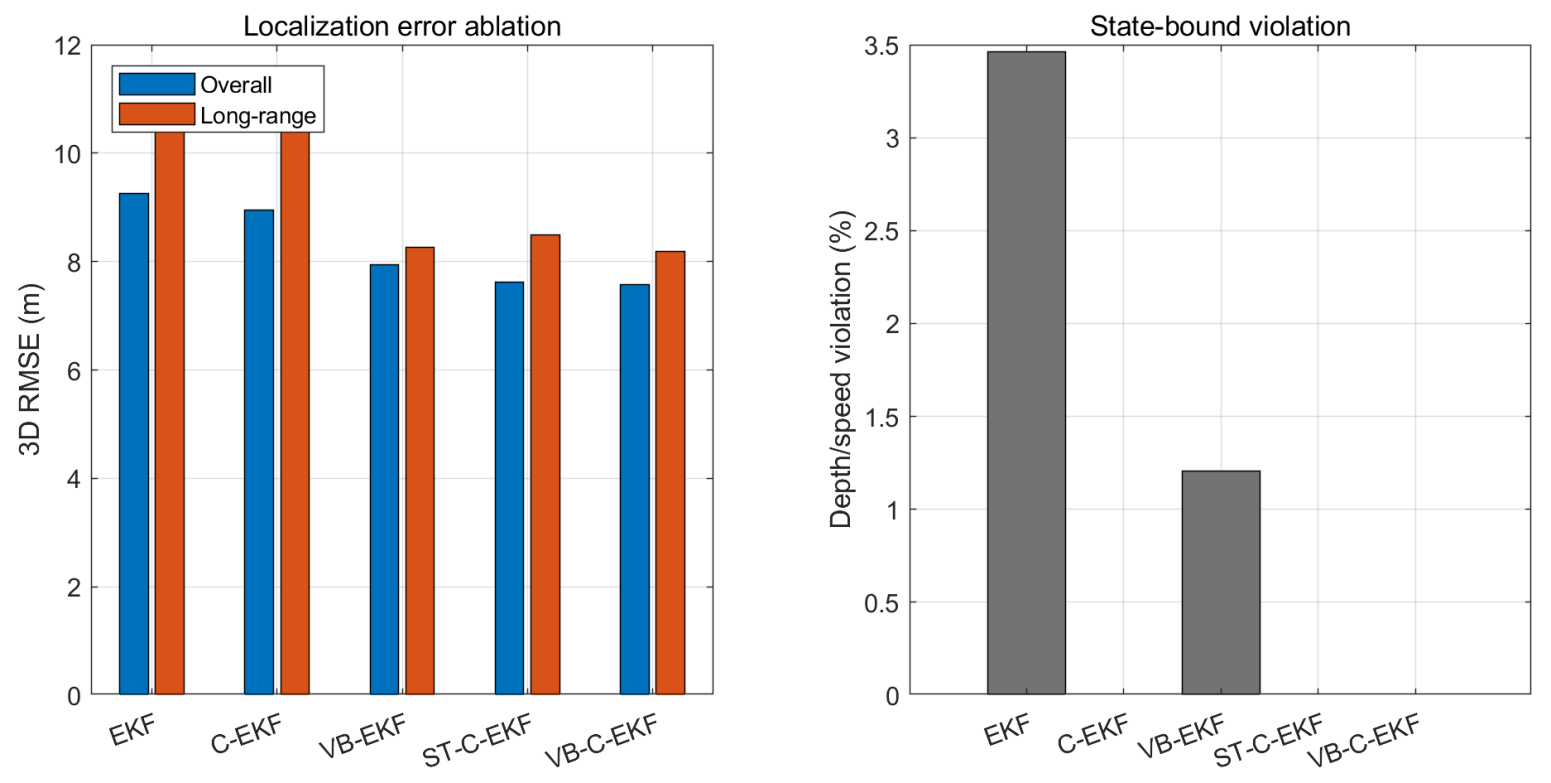
Main comparison results. The left panel compares the overall and long-range 3D RMSE values of EKF, C-EKF, VB-EKF, ST-C-EKF, and VB-C-EKF. The right panel compares the depth/speed bound violation rates.

**Figure 4:**
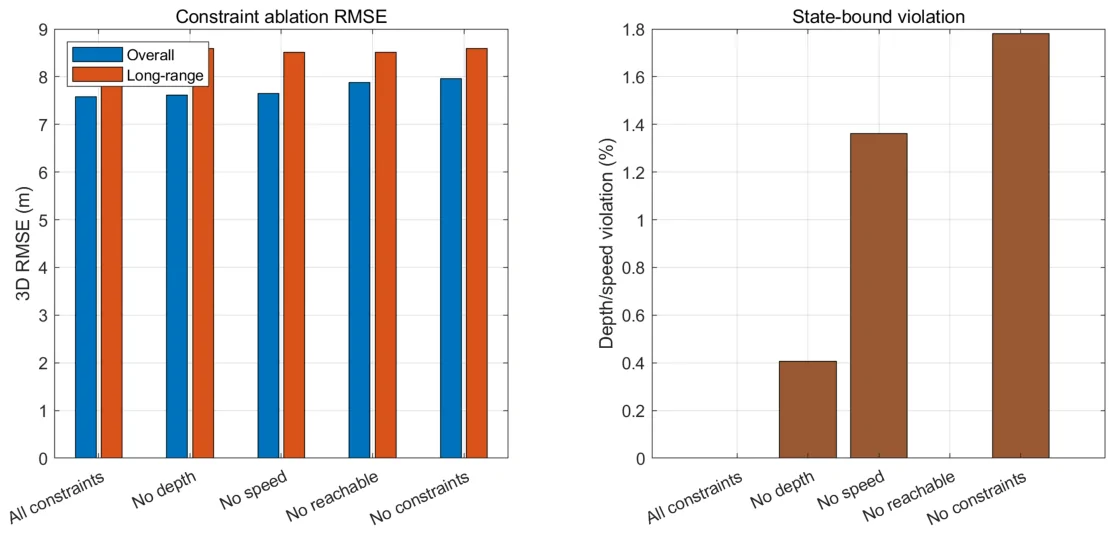
Constraint ablation results. The left panel shows overall and long-range 3D RMSE under different removed-constraint settings. The right panel shows the corresponding depth/speed bound violation rate.

**Figure 5:**
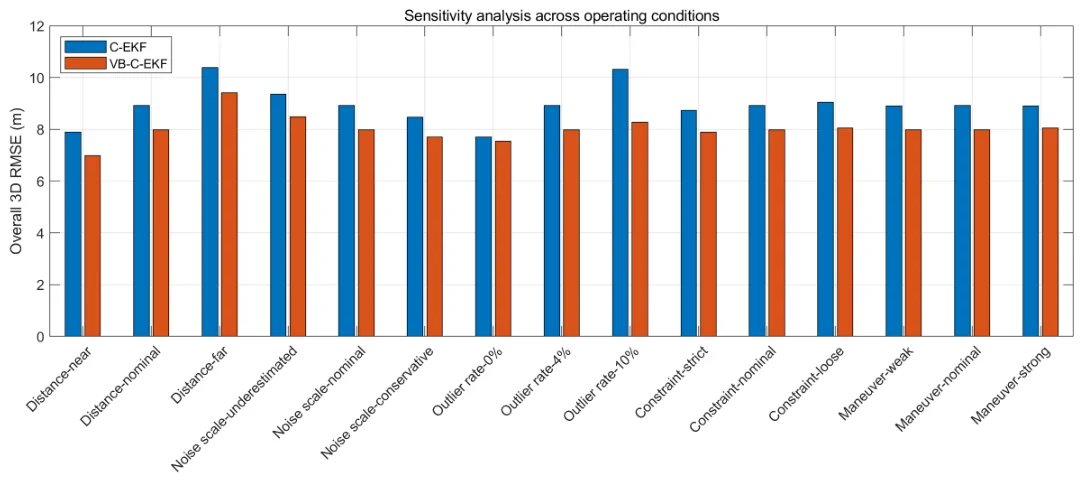
Sensitivity analysis across distance scale, nominal-noise mismatch, outlier rate, constraint strength, and maneuver intensity. VB-C-EKF consistently reduces the overall 3D RMSE compared with C-EKF.

The results are shown in Figure 1, Figure 2, Figure 3, Figure 4 and Figure 5 and Table 2, Table 3, Table 4 and Table 5. Because the filters do not receive the true time-varying covariance, the standard EKF has an overall 3D RMSE of 9.26m. The constrained EKF reduces this value to 8.95m and removes all depth/speed violations, but it still uses the mismatched nominal covariance. VB-EKF reduces the overall and long-range RMSE values to 7.94m and 8.26m, respectively, although it retains a 1.21% violation rate. The competitive ST-C-EKF baseline reaches 7.61m overall and 8.49m at long range with zero violations. VB-C-EKF gives the lowest overall RMSE, 7.57m, and the lowest long-range RMSE, 8.19m, while also maintaining zero violations. The difference between ST-C-EKF and VB-C-EKF in overall RMSE is small relative to their confidence intervals; the clearer VB advantage appears in long-range and depth errors.

Runtime was measured with MATLAB R2018b on a 64-bit Windows computer. It includes prediction, measurement update, adaptive or robust iterations, and constraint projection where applicable, but excludes measurement generation, metric calculation, and plotting. Each value is the average over 100×160 recursive updates.

Table 3 and Figure 2 quantify covariance consistency. The fixed-covariance EKF and C-EKF have mean NIS values of 11.19 and 11.10, far above the ideal measurement dimension of four, confirming overconfidence under covariance mismatch. VB-C-EKF reduces the mean NIS to 4.04 and the mean NEES from 13.07 to 6.79. The latter is close to, but slightly above, the upper ensemble bound of 6.698; therefore, the proposed method substantially improves consistency but is not claimed to be perfectly calibrated. Its NIS and NEES coverage rates are 60.6% and 43.8%, compared with 23.8% and 31.9% for ST-C-EKF. VB-C-EKF requires 0.225ms per update, approximately 2.3 times the C-EKF runtime but slightly less than the 0.234ms required by ST-C-EKF.

For the constraint-strength sensitivity experiment, the bathymetric depth constraint was kept unchanged, whereas the speed bound and the reachable-region slack were varied simultaneously. Specifically, the strict setting used $v_{\max}=6.6\;m/s$ and $\epsilon_{r}=1.0\;m$, the nominal setting used $v_{\max}=7.2\;m/s$ and $\epsilon_{r}=2.5\;m$, and the loose setting used $v_{\max}=8.5\;m/s$ and $\epsilon_{r}=6.0\;m$. The minimum operating depth $d_{\min}=3\;m$, bottom safety margin $d_{b}=3\;m$, and the reachable-region uncertainty scale were kept unchanged. All other simulation and filtering parameters were identical.

The constraint ablation in Table 4 shows that all three constraints are useful. Removing the reachable-region constraint produces the largest overall RMSE increase, indicating that the reachable-region constraint contributes most to improving overall position accuracy by suppressing unrealistic position jumps. Removing the speed constraint causes the largest bound-violation rate among single-constraint removals, indicating that the speed constraint contributes most to reducing the reported depth/speed bound violations, while removing the depth constraint mainly affects physical feasibility near the seabed or surface. The sensitivity analysis in Table 5 further shows that the VB-Constrained EKF remains stable across distance scaling, nominal noise mismatch, outlier probability, constraint strength, and maneuver intensity. The overall RMSE increases under farther target range, underestimated nominal covariance, and higher outlier rate, but VB-C-EKF consistently outperforms C-EKF and keeps the bound-violation rate at zero. The range-amplified localization condition remains visible: Long-range errors remain larger in most cases, and VB adaptation reduces but does not remove this physical effect.

## 4. Discussion

The constrained filter does not eliminate the physics of range-amplified angular error. Instead, it prevents the tracker from using physically impossible states to explain noisy long-range measurements. In shallow water, this is valuable because the bathymetry and operational envelope contain strong prior information. The depth constraint removes estimates above the surface or below the seabed. The speed constraint suppresses unrealistic velocity spikes caused by false alarms or angle wrapping. The reachable-region constraint reduces the line-of-sight ambiguity that is common in bearing-dominated tracking.

The method also improves track consistency. If a long-range bearing measurement has limited geometric information, the posterior covariance remains large in weakly observable directions. The Mahalanobis mean projection uses this anisotropic uncertainty when correcting an infeasible state, while the conservative covariance regularization avoids treating repeated boundary contact as a highly accurate measurement.

The VB component is most useful when the range-dependent noise law is only approximate. If the sonar has been well calibrated and $R_{k}$ is available from reliable online signal-quality estimation, the standard constrained EKF is sufficient and computationally simpler. If isolated heavy-tailed outliers dominate, ST-C-EKF provides a competitive robust alternative. In the simulated continuously varying covariance, however, VB-C-EKF gives better long-range accuracy, NIS coverage, and runtime than ST-C-EKF because it recursively carries information about the observation covariance instead of estimating only an instantaneous residual weight.

Several engineering details are important. First, angular residuals must be normalized. Second, range-dependent covariance should be updated at each time step according to the predicted geometry. Third, physical constraints should be used only when the environmental information is reliable. If the seabed map or target speed bound is uncertain, a soft-constraint implementation is safer. Fourth, the present study assumes valid active-sonar range–bearing–elevation–Doppler measurements; missed detections, false alarms, and data-association errors remain to be addressed.

The present validation is simulation-based. The small-target difficulty is represented through weak maneuverability, uncertain range-dependent measurements, outliers, and shallow-water constraints, but the acoustic target strength, echo waveform, detection probability, missed detection, and data-association process are not explicitly modeled. Future work should combine the proposed estimator with realistic active-sonar detection front ends, high-fidelity acoustic propagation simulation, moving or multi-station platforms, and field or tank-test data.

## 5. Conclusions

This paper formulated active-sonar-based underwater target tracking in complex shallow water as a constrained nonlinear Kalman filtering problem. The model accounts for range-amplified angular error through range-dependent measurement covariance and nonlinear sonar geometry. Bathymetric depth, speed, and reachable-region constraints were incorporated through sequential local Mahalanobis mean projection and a conservative, implementation-consistent covariance correction. Under unknown time-varying measurement noise, VB-C-EKF achieved an overall 3D RMSE of 7.57±0.25m, compared with 8.95±0.20m for C-EKF, 7.94±0.37m for VB-EKF, and 7.61±0.20m for the competitive ST-C-EKF baseline. It maintained zero depth/speed violations, reduced the mean NIS and NEES to 4.04 and 6.79, and required 0.225ms per update on the test computer. The evidence is simulation-based and supports improved accuracy and consistency under the tested conditions rather than universal superiority. Future work should evaluate detection uncertainty, data association, moving or multi-station platforms, and field or tank-test data.

## Figures and Tables

**Table 1:** Constrained EKF for Active-Sonar Underwater Target Tracking in Shallow Water.

Step	Operation
1	Predict ${\hat{x}}_{k}^{-}$ and $P_{k}^{-}$ using the weak-maneuver dynamic model.
2	Compute the predicted target-platform range and construct nominal range-dependent $R_{0,k}$.
3	If the observation noise is uncertain, update ${\hat{R}}_{k}$ by the VB iterations in Equations (46)–(51).
4	Evaluate $h({\hat{x}}_{k}^{-})$, the Jacobian $H_{k}$, innovation $\nu_{k}$, and innovation covariance $S_{k}$.
5	Perform EKF update to obtain unconstrained ${\hat{x}}_{k}^{u}$ and $P_{k}^{u}$.
6	Test depth, speed, and reachable-region constraints.
7	Linearize violated nonlinear constraints and form the active constraint matrix.
8	Apply the sequential local Mahalanobis mean projection in Equation (40), then update the covariance using Equations (41)–(43).
9	Output ${\hat{x}}_{k}^{+}$, $P_{k}^{+}$, and adaptive ${\hat{R}}_{k}$.

**Table 2:** MATLAB Monte Carlo results. Values are reported as the mean ± 95% confidence-interval half-width calculated from 100 run-wise metric values.

Metric	EKF	C-EKF	VB-EKF	ST-C-EKF	VB-C-EKF	Unit
Overall 3D position RMSE	9.26 ± 0.22	8.95 ± 0.20	7.94 ± 0.37	7.61 ± 0.20	7.57 ± 0.25	m
Overall horizontal RMSE	7.74 ± 0.25	7.51 ± 0.23	6.85 ± 0.40	6.43 ± 0.22	6.48 ± 0.27	m
Overall depth RMSE	4.98 ± 0.17	4.79 ± 0.15	3.87 ± 0.14	3.99 ± 0.13	3.83 ± 0.13	m
Long-range 3D position RMSE	10.68 ± 0.49	10.44 ± 0.46	8.26 ± 0.40	8.49 ± 0.37	8.19 ± 0.39	m
Long-range horizontal RMSE	8.49 ± 0.50	8.47 ± 0.50	6.52 ± 0.41	6.71 ± 0.39	6.52 ± 0.41	m
Long-range depth RMSE	6.18 ± 0.37	5.82 ± 0.31	4.76 ± 0.34	4.93 ± 0.30	4.66 ± 0.31	m
Final-time 3D position RMSE	9.61 ± 1.00	9.49 ± 0.96	7.73 ± 0.77	7.88 ± 0.82	7.60 ± 0.74	m
Depth/speed bound violation rate	3.46 ± 0.48	0.00 ± 0.00	1.21 ± 0.42	0.00 ± 0.00	0.00 ± 0.00	%

**Table 3:** Filter consistency and computational cost. Coverage is the percentage of time steps for which the ensemble-averaged statistic lies inside its two-sided 95% chi-square interval.

Filter	Mean NIS	NIS Coverage (%)	Mean NEES	NEES Coverage (%)	Time/Update (ms)
EKF	11.19	0.6	13.60	0.0	0.076
C-EKF	11.10	0.6	13.07	0.0	0.100
VB-EKF	4.04	60.6	7.11	43.8	0.196
ST-C-EKF	4.79	23.8	7.08	31.9	0.234
VB-C-EKF	4.04	60.6	6.79	43.8	0.225

**Table 4:** Constraint Ablation for VB-Constrained EKF. A value of 1 means that the corresponding constraint is enabled.

Case	Depth	Speed	Reach	Overall RMSE	Long-Range RMSE	Bound Vio.
All constraints	1	1	1	7.58	8.50	0.00
No depth	0	1	1	7.61	8.59	0.41
No speed	1	0	1	7.65	8.51	1.36
No reachable	1	1	0	7.88	8.51	0.00
No constraints	0	0	0	7.96	8.59	1.78

**Table 5:** Sensitivity Analysis Under Different Range, Noise, Outlier, Constraint, and Maneuver Conditions.

Factor	Level	C-EKF	VB-C-EKF	VB-C Long	Bound Vio.
Distance	near	7.90	6.99	8.10	0.00
Distance	nominal	8.91	7.98	8.40	0.00
Distance	far	10.39	9.41	8.40	0.00
Noise scale	underestimated	9.35	8.48	8.63	0.00
Noise scale	nominal	8.91	7.98	8.40	0.00
Noise scale	conservative	8.47	7.70	8.17	0.00
Outlier rate	0%	7.70	7.55	8.54	0.00
Outlier rate	4%	8.91	7.98	8.40	0.00
Outlier rate	10%	10.31	8.28	9.45	0.00
Constraint	strict	8.72	7.89	8.39	0.00
Constraint	nominal	8.91	7.98	8.40	0.00
Constraint	loose	9.04	8.04	8.40	0.00
Maneuver	weak	8.91	7.97	8.40	0.00
Maneuver	nominal	8.91	7.98	8.40	0.00
Maneuver	strong	8.91	8.05	8.46	0.00

## Data Availability

The data supporting the findings of this study are available from the corresponding author upon reasonable request.

## Abbreviations

The following abbreviations are used in this manuscript:

AIS	Automatic Identification System
C-EKF	Constrained Extended Kalman Filter
EKF	Extended Kalman Filter
IMM	Interacting Multiple Model
NEES	Normalized Estimation Error Squared
NIS	Normalized Innovation Squared
RMSE	Root Mean Square Error
ST-C-EKF	Student’s-*t* Constrained Extended Kalman Filter
UKF	Unscented Kalman Filter
VB	Variational Bayesian
VB-C-EKF	Variational Bayesian Constrained Extended Kalman Filter
